# Evaluation of Nanocurcumin Effects on Depressive‐Like Behaviors in Rats and Determination of Serum BDNF and Serotonin Levels

**DOI:** 10.1002/brb3.70320

**Published:** 2025-02-19

**Authors:** Mahsa Hadipour Jahromi, Hasti Charousaei, Ali Charousaei

**Affiliations:** ^1^ Department of Pharmacology, Tehran Medical Science Branch Islamic Azad University Tehran Iran; ^2^ Maternal, Fetal and Neonatal Health Research Center, Institute of Family Health Tehran University of Medical Sciences Tehran Iran

**Keywords:** BDNF, depression, nanocurcumin, rat, serotonin

## Abstract

**Introduction:**

Major depressive disorder (MDD) is a highly prevalent psychiatric condition worldwide, and it is the leading cause of disability globally. Turmeric, an aromatic perennial herb widely used in traditional Asian medicine and cuisine, contains curcumin, which has several biological effects, including a pseudoantidepressant effect. However, curcumin's low bioavailability limits its effectiveness. This study evaluated nanocurcumin (NCUR) effects on depressive‐like behaviors and examined serum BDNF and serotonin levels in a chronic stress model in rats. Behavioral assessments and biochemical indicators elucidated NCUR's antidepressant‐like properties.

**Methods:**

In this experimental study, 30 adult male rats were randomly divided into six groups and exposed to unexpected chronic mild stress (UCMS). The groups included: control (CG), stress control (SCG), fluoxetine (FLU) treatment (20 mg/kg), and three NCUR doses (20, 40, and 80 mg/kg). Before UCMS exposure, rats underwent a sucrose preference test (SPT). Depressive behaviors were then assessed using the open field test (OFT), forced swimming test (FST), and tail suspension test (TST) on days 27 and 28. Blood samples were collected on day 28 to measure serum Brain derived neurotrophic factor (BDNF) and serotonin levels using enzyme‐linked immunosorbent assay (ELISA).

**Results:**

NCUR treatment significantly alleviated depressive‐like behaviors in stressed rats. The sucrose preference index of the SCG decreased after 26 days of stress, while NCUR (all doses) and FLU reversed this effect. In the FST and TST, immobility time was significantly reduced in the NCUR and FLU groups compared to the SCG (*p* < 0.05). The OFT also showed that the SCG had significantly fewer crossings compared to treated groups (*p* < 0.05). Additionally, NCUR treatment significantly increased serum BDNF and serotonin levels compared to the SCG.

**Conclusions:**

This study demonstrates that NCUR exerts antidepressant‐like effects, improving depressive behaviors and increasing BDNF and serotonin levels in rats exposed to chronic stress. NCUR may offer a promising alternative for the treatment of MDD.

## Graphical abstract

Nanocurcumin significantly alleviates depressive‐like behaviors in rats exposed to unexpected chronic mild stress. It enhances serum BDNF and serotonin levels, suggesting its potential as an effective antidepressant. Behavioral improvements were evident through various tests, highlighting its promise in treating depression.

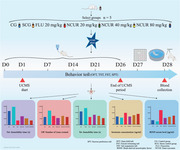

## 1 Introduction

Depression or major depressive disorder is the most common psychiatric disorder worldwide (James et al. 2018). Among the top 10 causes of disability (including migraines, falls, hearing loss, diabetes, anxiety disorders, alcohol use disorders, chronic lung conditions, iron deficiency anemia, neck and back pain, and depression), depression represents the most prevalent cause (Smith 2014). Depressive mood, anhedonia, sleep disturbances, appetite disturbances, and feelings of guilt or worthlessness characterize it. About 5–17% of the population worldwide (about 300 million people) have suffered from this disorder at least once in their life (World Health Organization 2017). A crucial aspect of depression is that, despite its high prevalence, it is either undiagnosed or untreated; the longer the disorder is untreated, the greater the likelihood that it will become resistant to treatment (Kraus et al. 2019). In light of this, it is necessary to look for other treatments due to a lack of response or reduced response to pharmacotherapeutic treatments.

In recent years, BDNF has received substantial attention as it has been shown to reverse or inhibit stress‐induced apoptosis, is essential for neurogenesis, and plays a significant role in neuroplasticity (Jiang and Salton 2013). The level of BDNF is significantly reduced by stressful factors and increases with various antidepressant interventions (Pittenger and Duman 2008). Depression generally results in a decrease in the plasma level of BDNF (Nobis et al. 2020). Studies have examined the effect of drugs and herbs on depression by examining BDNF levels.

Unexpected chronic mild stress (UCMS) involves rodents exposed to a wide range of mild stressors that cannot cause tangible reactions. Therefore, these stressors do not cause physical pain, are chronic, and occur for consecutive weeks when they are not expected. The examined rodents are expected to develop anhedonia after a couple of weeks of exposure to this model, which can be evaluated with tests such as the sucrose preference test (SPT) or the cookie test (Planchez et al. 2019). This model has several benefits, including similarity to human depression, predictability, helping choose the best antidepressants, measuring anhedonia, and identifying depression risk factors (Hao et al. 2019).

Several tests are used to investigate depression‐like behaviors in rodents. Several tests are used to assess frustration, including forced swimming tests (FSTs) and tail suspension tests (TSTs), open field tests (OFTs), and feeding suppression tests that can measure anxiety. Rodents’ indifference is assessed by disrupting nesting and grooming with splash tests and disrupting their coats. As previously mentioned, sucrose preference and cookie tests assess anhedonia (Planchez et al. 2019).

Curcumin is a polyphenol compound found in turmeric (*Curcuma longa*), with a relatively low molecular weight. Turmeric has a rich historical heritage in Southeast Asia, where it is widely employed as a spice, coloring agent, food preservative, and traditional medicinal component (Ramaholimihaso et al. 2020). Curcumin offers a wide range of therapeutic qualities, including anticancer, antiviral, antifungal, antioxidant, antiangiogenic, and anti‐inflammatory effects. (Rahmani et al. 2018). Previously, the impact of curcumin on several neurological and psychological disorders was investigated. (Bhat et al. 2019). Experimental studies have also demonstrated that curcumin can have a substantial impact on stress‐induced depression and noninduced depression in mice models. (Abd‐Rabo et al. 2019; Hurley et al. 2013; Wang et al. 2014; Wu et al. 2021; Zhang et al. 2014, 2012; Faucher et al. 2022). Also in clinical studies, it was demonstrated that curcumin can reduce anxiety significantly (Fathi et al. 2024).

Curcumin has been studied in numerous clinical trials for its antidepressant‐like characteristics. According to Lopresti et al. (2014), in a double‐blind, randomized trial, 500 mg curcumin capsules were administered for 8 weeks to 56 subjects. The results showed that curcumin improved mood‐related symptoms in the second 4 weeks compared to the placebo. Also, curcumin significantly affected patients with atypical depression. Sanmukhani et al. (2014) suggested that curcumin could be a safe and effective treatment for depression without suicidal thoughts. A double‐blinded cross‐over trial by Esmaily et al. (2015) found that curcumin effectively reduced depression and anxiety in obese patients. Some clinical trials have shown that curcumin has significant depression‐reducing properties in combination with saffron (Lopresti and Drummond 2017), ellagic acid which enhances curcumin absorption (Bergman et al. 2013), and curcuminoids‐piperine (Panahi et al. 2015) as an add on therapy to standard antidepressant therapy.

Curcumin, despite its known therapeutic effects, suffers from poor solubility, rapid metabolism, and limited absorption, which greatly restricts its clinical effectiveness, thus its weak pharmacodynamics makes it less effective as a medicinal agent (Moniruzzaman and Min 2020). Due to its high sensitivity to absorption by the reticuloendothelial system, curcumin cannot maintain its therapeutic level, resulting in low systemic bioavailability (Rabiee et al. 2018). Standard curcumin formulations require high doses to reach therapeutic levels, which is not always feasible and may still fail to maintain sustained bioactivity within the body. Lao et al. (2006) found traces of curcumin and its metabolites in humans' plasma and urine after consuming 12 g/day of food. Therefore, the therapeutic threshold can be reached by consuming about 12–20 g of curcumin per day (Basniwal et al. 2011).

With the advent of nano therapy, many ways to enhance the biodistribution of curcumin have been proposed (Flora et al. 2013). In contrast to curcumin standard formation, NCUR's nanoformulation allows for better dispersal in aqueous environments, leading to increased systemic availability and retention. (Flora et al. 2013) This improved pharmacokinetic profile means NCUR can achieve effective concentrations at lower doses, making it a more efficient option for therapeutic use. In this way, curcumin is transformed from a food spice to a clinical drug that maintains its threshold level and provides a balanced dose. Curcumin delivered at the nanoscale has also been shown to significantly increase body retention, circulation, and mean residence time (Flora et al. 2013). The effectiveness of NCUR allows it to reach organs inaccessible to curcumin. A study on rats by Tsai et al., compared to the distribution pattern of curcumin and NCUR in different organs after intravenous injection, shows that the liver and kidney had higher concentrations of curcumin. In comparison, NCUR was significantly increased in the spleen, lung, liver, brain, and kidney (Tsai et al. 2011). Moreover, while standard curcumin standard curcumin has shown promise in reducing neuroinflammation and supporting cognitive and mood‐related biomarkers associated with depression, such as BDNF, its limited absorption reduces its impact. NCUR, however, is engineered for enhanced absorption, allowing it to reach the brain more effectively (Tsai et al. 2011). This improved bioavailability enables NCUR to exert a stronger influence on critical biomarkers like BDNF, nuclear factor kappa B (NF‐κB), and oxidative stress markers, which are linked to neurogenesis, inflammation regulation, and mood enhancement (Kodali et al. 2023). By modulating inflammatory cytokines such as TNF‐α and IL‐6 and boosting BDNF signaling, NCUR shows a stronger impact on neuroplasticity and cognitive function, positioning it as a promising therapeutic approach for mood disorders and cognitive impairments (Kodali et al. 2023). Thus, the nanoformulation of curcumin not only addresses the limitations of standard curcumin but also opens new avenues for its application in treating complex neuropsychiatric conditions.

This study aimed to examine the antidepressant effects of NCUR compared to fluoxetine (FLU) through behavioral tests and the effect of NCUR on BDNF and serotonin levels in blood serum.

## 2 Material and Methods

### 2.1 Experimental Animals

A total of 30 adult males and 8‐week‐old rats (weighing about 250 g) were relocated to the animal facility of the Pharmacology Research Center at the Faculty of Medical Sciences of Islamic Azad University, Tehran, Iran. The animals were maintained at a temperature range of 20–30°C and optimal conditions in terms of humidity and light and were provided unrestricted access to food and water throughout the study period. All animals were housed in a cage with a light–dark cycle of 12 h each for a duration of 1 week. This was done to familiarize the animals with the circumstances of the animal house. All laws and ethics were followed by the Ethics Research Code of the Islamic Azad University of Iran and approved by the faculty (code: IR.IAU.PS.REC.1400.494).

### 2.2 Study Design

In this experimental study, oral solutions (with olive oil as the solvent) were prepared from NCUR (Exir Nano Sina Co., Iran) and FLU (Tehran Darou Co., Iran) for behavioral tests.

After 1 week of adaptation, the animals were randomly divided into six groups (*n* = 5) and received oral gavage treatment for 26 days for the UCMS: the control and the stress control (SCG) with olive oil; 10 mg/kg FLU (Huang et al. 2019); 20 mg/kg NCUR; and 40 mg/kg NCUR, and 80 mg/kg NCUR. Fluoxetine at a dose of 10 mg/kg was selected based on its established efficacy in reducing depressive‐like behaviors in rodent models of chronic stress‐induced depression, commonly used in preclinical studies for its antidepressant effects without severe side effects (Huang et al. 2019). All rats had ad libitum access to food and water. The control (CG) group was isolated in a distinct and uninterrupted location, whereas the remaining groups of rats were exposed to UCMS stressors. All rats were subjected to a baseline SPT before the UCMS test.

**FIGURE 1:**
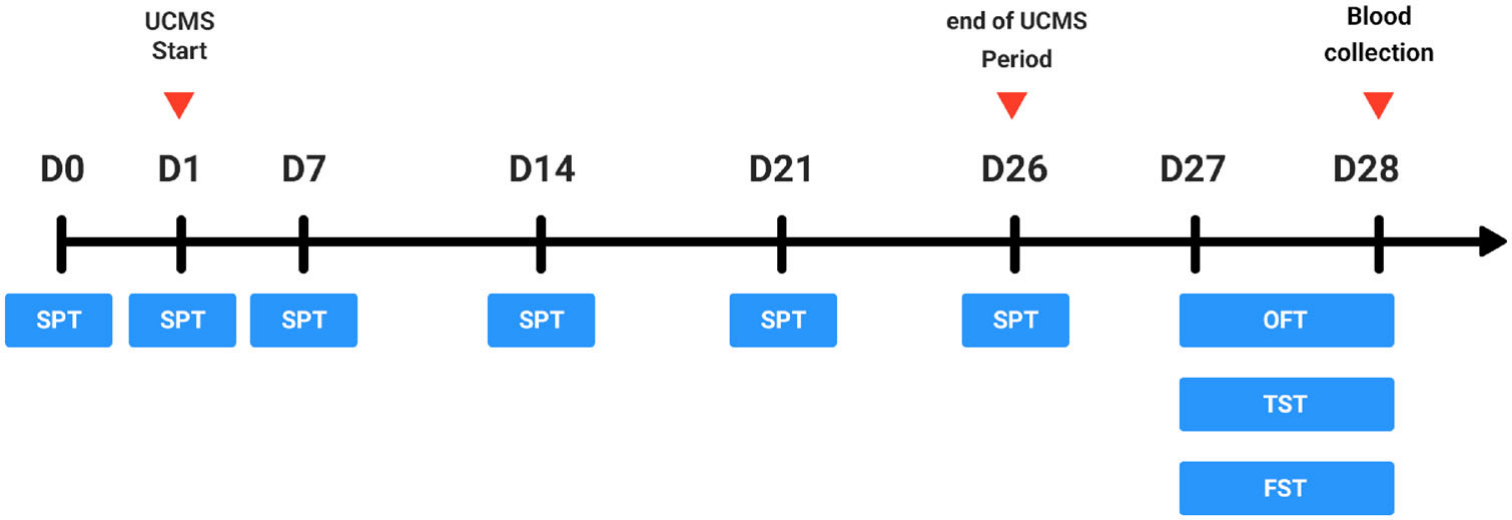
General schedule of behavioral tests in 28 days (adapted from Huang et al. 2019).

The first SPT test was performed on day 0 and the rest on the 7th, 14th, 21st, and 26th days. After completing the UCMS period, other behavioral tests, including OFT, TST, and FST, were performed, and finally, blood samples were collected to evaluate serotonin and BDNF levels. The general schedule of behavioral tests is shown in Figure 1.

### 2.3 Unexpected Chronic Mild Stress

UCMS is one of the most valid and realistic models of depression that focuses on anhedonia. This method exposes animals to a series of mild stressors (Zavvari and Karimzadeh 2015). Some of the stressful factors performed to induce depressive‐like behavior in this study include restriction of movement (60 –150 min), deprivation of water or food or both (6 –15 h), a cage with wet sawdust (15–19 h), cage without sawdust (15–24 h), forced swimming (30 min), tilted cage (15–24 h), and reversal of the light–dark cycle (24 h). Among the mentioned items, at least two stressors have been implemented daily for the rats. The time of exposure to these factors can be between 2 weeks to several months. In this study, the test duration was 26 days, and after this period the stressors were stopped and other behavioral tests were performed on the rats.

### 2.4 Sucrose Preference Test

In order to assess anhedonia, a SPT was performed. During the trial, rats were treated with 1% (w/w) sucrose solution and pure water once a week for 1 h (Huang et al. 2019). The two preweighted bottles were given to the rats. Afterward, the bottles were weighed again, and the difference in weight between each bottle was considered the rats' intake (Zavvari and Karimzadeh 2015). The first SPT was conducted before the start of the UCMS phase. The rest of these tests were performed on the study's 7th, 14th, 21st, and 26th days. The rats were subjected to a 24‐h period of food and water deprivation before the SPT was conducted. Before the test, both sucrose and pure water were provided ad libitum as a pretest for 24 h.

The ratio of sucrose preference was calculated using the following equation:

$$\mathrm{Sucrose}\;\mathrm{preference}\;\%\;=\frac{\mathrm{sucrose}\;\mathrm{intake}}{\mathrm{sucrose}\;\mathrm{intake}\;+\;\mathrm{water}\;\mathrm{intake}}\times 100\;\%.$$

SPTs were performed every week following a 24‐h period of food and water deprivation.

### 2.5 Forced Swimming Test

FST is one of the valid tests to check rodents' frustration levels. In this test, the rats are placed individually into a cylinder (25 cm tall and 16 cm in diameter) filled with water to a level of 20 cm at a temperature of 25 ± 1°C (about two‐thirds of the container is filled with water so that the rat's foot does not reach the bottom of the container and cannot escape using its legs and tail) (Zavvari and Karimzadeh 2015). FST was conducted for all animals for 6 minutes within the 27th and 28th days. A video of FST behavior was recorded and immobility time was calculated.

### 2.6 Open Field Test

To detect spontaneous locomotor activity, OFT evaluated the rats. Each rat was placed at the center or corner of a wooden box (50  long × 50  wide × 25 cm high) for 2 min for adaptation, and locomotor activity was assessed for 4 min. The open field apparatus was divided into 25 equal squares. Test sessions were recorded to analyze the bouts (the number of segments crossed with all four paws) for each of the rats individually. Mice with a depression background usually have less mobility in this test and rarely stand on their paws (Zavvari and Karimzadeh 2015).

### 2.7 Tail Suspension Test

This test is similar to the FST test for assessing rodents' depression. The test is performed by hanging a rat from the middle two‐thirds of its tail from a distance of 50 cm above ground level. The test starts with a sharp movement of the mouse. Following the intense movement of the mouse, it becomes completely motionless and unresponsive. Immobility time will be recorded in a 4 minutes period. The total duration of the hanging procedure will be 6 min, with the first 2 minutes excluded to allow the animal to familiarize itself with the environment (Zavvari and Karimzadeh 2015).

### 2.8 Determination of BDNF and Serotonin Levels

The rats were anesthetized and then euthanized by decapitation at the end of the experiment. The blood samples were collected immediately after the end of the trial. The serum concentration of BDNF and serotonin was determined by enzyme‐linked immunosorbent assay (ELISA) testing kits. Specifically, the BDNF ELISA kit (catalog number MBS355435) and the serotonin (5‐HT) ELISA kit (catalog number MBS723181), both obtained from MyBioSource (San Diego, CA, USA), were utilized. Assays were conducted following the manufacturer's protocols to ensure accuracy and reproducibility.

### 2.9 Statistical Analysis

Statistical analysis was performed using one‐way analysis of variance (ANOVA) and Duncan's multiple‐comparison test using SPSS version 26; *p* < 0.05 was considered to be statistically significant.

## 3 Results

### 3.1 Effects of NCUR on the SPT

**FIGURE 2:**
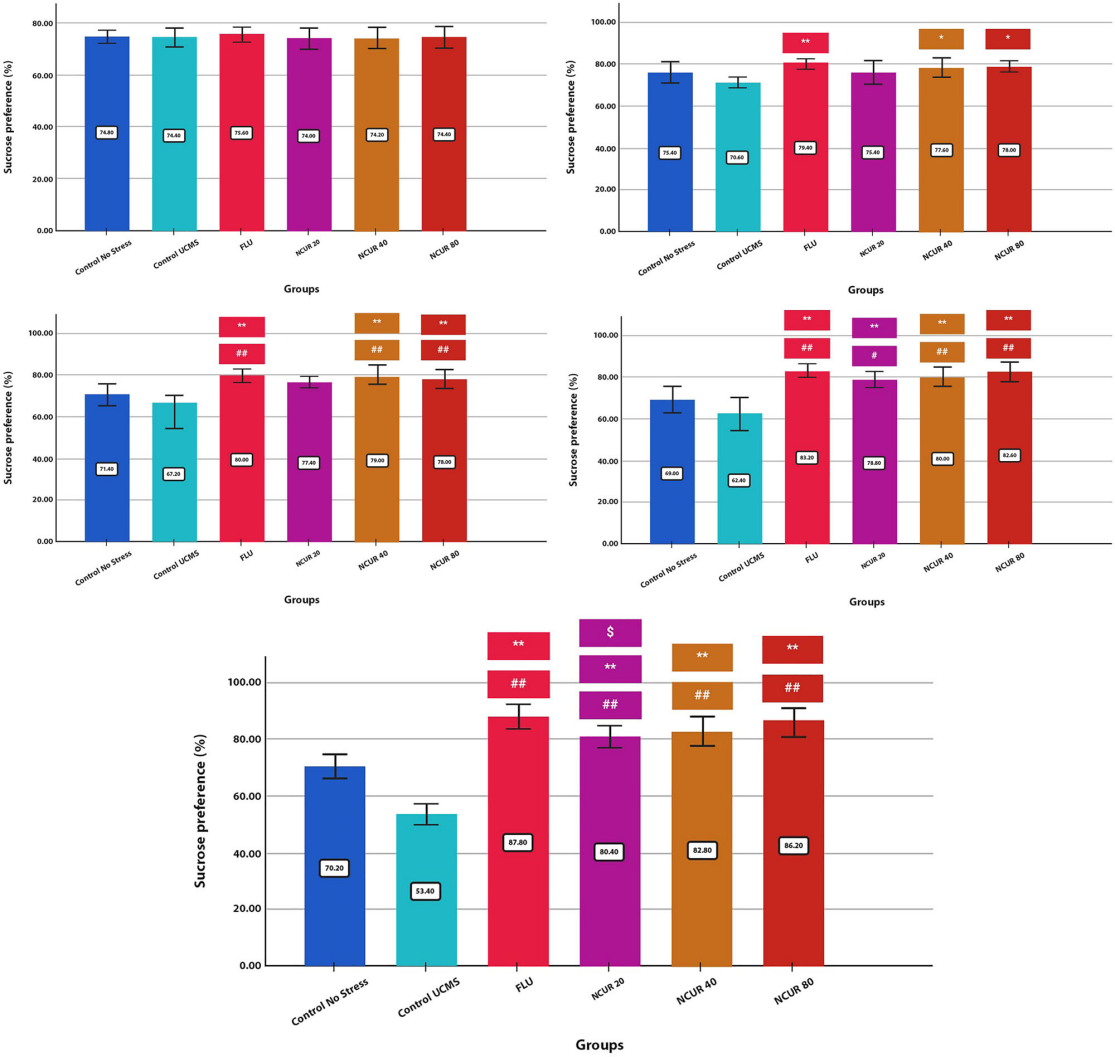
The effect of different doses of NCUR compared to FLU in the SPT. Each group (*N* = 5) was subjected to 26 days of oral gavage. Before the start of the UCMS period, there was no difference between the groups (day 0). After the completion of the UCMS interval, a significant difference was observed between the groups (day 26) (*p* = 0.000).

The effects of NCUR and FLU on the sucrose preference index during the experiment, after 26 days of treatment, are presented in Figure 2.

Stress paradigms are being employed to study the field of depression. Typically, researchers subject individuals to uncontrollable stress to assess their reaction to antidepressant medications. UCMS induces anhedonia, characterized by elevated plasma corticosterone levels and reduced sucrose preference (Huang et al. 2019). Depression can be caused by several unpredictable or inescapable stressors, including physiological changes, such as a reduction in sexual aggression and locomotor activity (Katz et al. 1981). The UCMS is a validated model of depression in rodents, simulating several symptoms of depression in humans (Mineur et al. 2006). Consequently, we investigated the antidepressant‐like effects of NCUR utilizing a UCMS model. Our results demonstrated that the administration of NCUR reversed the UCMS‐induced decrease in the sucrose preference index.

Day 0: There were no significant differences between the groups in terms of sucrose preference index during the baseline phase (*p* = 0.960).

Day 7: There was no significant difference between NCUR and the CG. However, there was an increase in sucrose craving compared with the SCG (P80 = 0.002, P40 = 0.003, P20 = 0.032).

Day 14: There was a significant difference between all three doses of NCUR, the CG (P80 = 0.004, P40 = 0.002, P20 = 0.013) and the SCG (*p* = 0.000).

Day 21: All three doses of NCUR demonstrated a significant increase in the sucrose preference index compared to the CG (P80 = 0.000, P40 = 0.001, P20 = 0.002), and the SCG (*p* = 0.000).

Day 26: The doses of 20, 40, and 80 mg/kg of NCUR were significantly different compared to the CG (*p* = 0.000) and the SCG (*p* = 0.000); while among the doses of NCUR, 20 and 40 mg showed a significant difference in the preference of rats for water containing sucrose compared with FLU (P40 = 0.034, P20 = 0.003).

Day 0 (upper left), Day 7 (upper right), Day 14 (middle left), Day 21 (middle right), and Day 26 (lower row). *p* < 0.05 is indicated by #, and *p* < 0.001 is indicated by ## (comparing the CG with other groups). *p* < 0.05 is shown with * and *p* < 0.001 with **(comparing the SCG with other groups). *p* < 0.05 is shown with $ and *p* < 0.001 is shown with $$ (comparison of FLU with other groups)

Also, after 26 days, the sucrose preference index in the SCG was drastically lower than in the CG.

The behavioral data reported here are similar to those found in previous studies investigating the effects of chronic medication, such as FLU, used in depression treatment (Abd‐Rabo et al. 2019).

### 3.2 Effects of NCUR on Immobility Time in the FST

**FIGURE 3:**
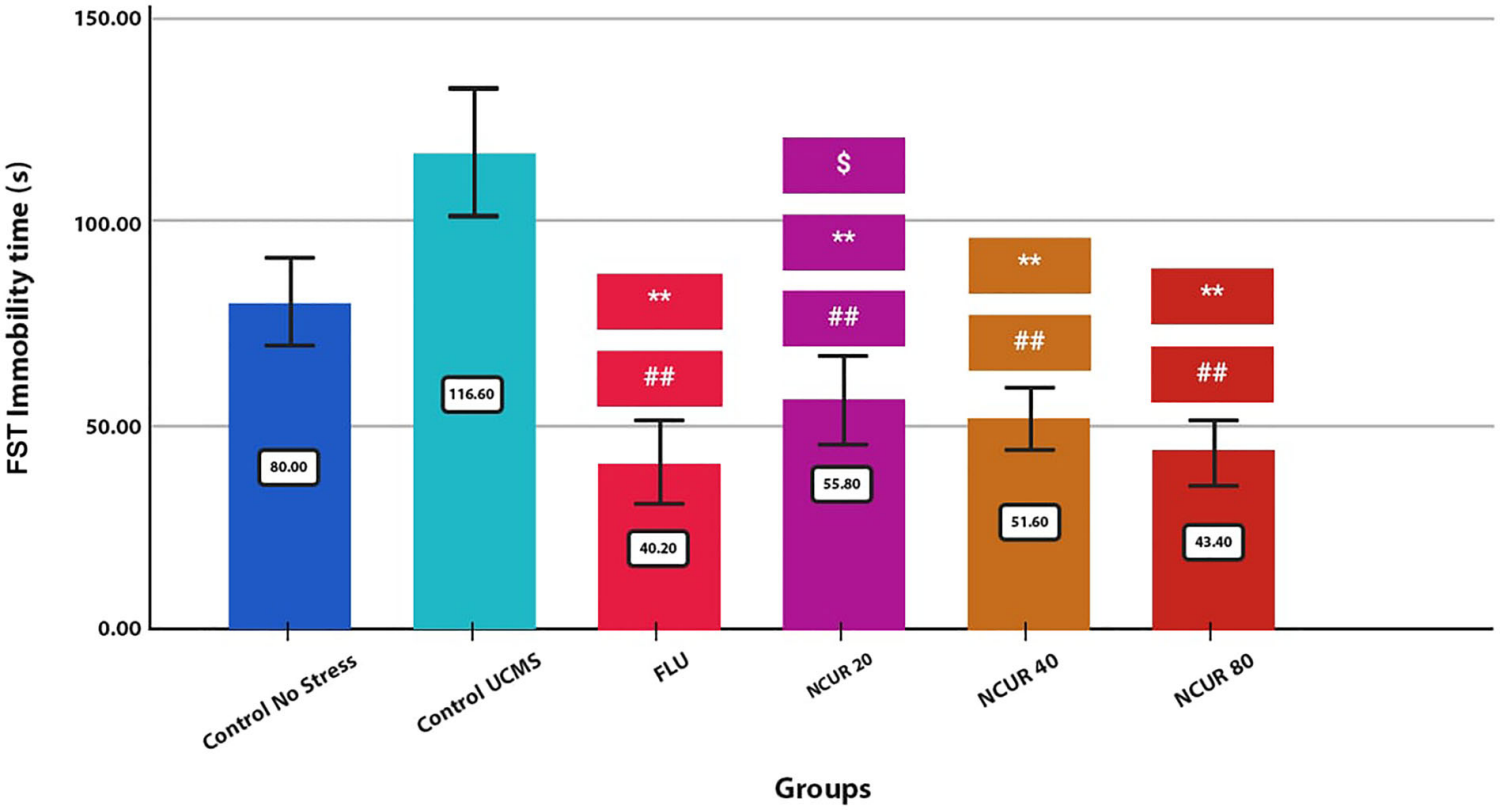
The effect of different doses of NCUR compared to FLU in the FST. Each group (*N* = 5) was subjected to 26 days of oral gavage. *p* < 0.05 is indicated by #, and *p* < 0.001 is indicated by ## (comparing the CG with other groups). *p* < 0.05 is shown with * and *p* < 0.001 with **(comparing the SCG with other groups). *p* < 0.05 is shown with $, and *p* < 0.001 is shown with $$ (comparison of FLU with other groups).

The effects of oral administration of NCUR (20, 40, and 80 mg per kg) and FLU on immobility time in the FST are shown in Figure 3. There is widespread use of the FST test to screen for potential depressants with antidepressant properties. Previously, modified FSTs were used in rats to differentiate between classes of antidepressant drugs depending on their activity levels, such as climbing, swimming, and diving (Detke and Lucki 1995). In the FST, selective norepinephrine‐targeting antidepressants increase climbing activity, whereas serotonin neurotransmission‐related medications promote swimming behavior (Bogdanova et al. 2013).

The results of the present study confirmed the hypothesis that NCUR has antidepressant‐like effects in the FST (*p* = 0.000). All of the doses of NCUR showed significant differences in shortening the immobility time compared to the CG (*p* = 0.000) and the SCG (*p* = 0.000). Among the three doses of NCUR compared to FLU, only the 20‐mg dose showed a significant difference in reducing immobility time (P20 = 0.010).

### 3.3 Effects of NCUR on the TST

**FIGURE 4:**
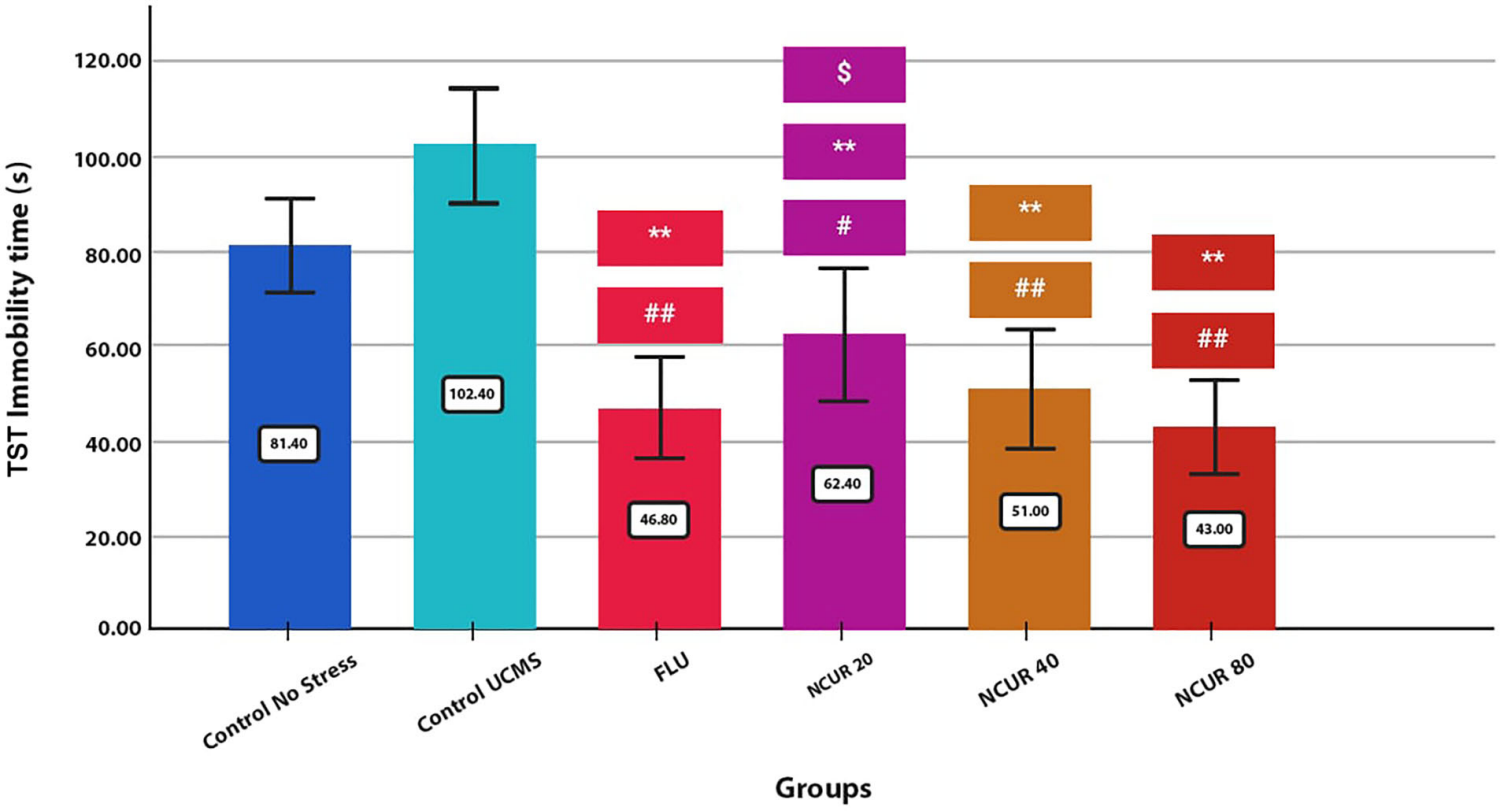
The effect of different doses of NCUR compared to FLU in the TST. Each group (*N* = 5) was subjected to 26 days of oral gavage. *p* < 0.05 is indicated by #, and *p* < 0.001 is indicated by ## (comparing the CG with other groups). *p* < 0.05 is shown with * and *p* < 0.001 with **(Comparing the SCG with other groups). *p* < 0.05 is shown with $, and *p* < 0.001 is shown with $$ (comparison of FLU with other groups).

The TST results confirmed the effects of all NCUR doses on decreasing the immobility time of the rats (Figure 4). There was a significant difference between all three doses of NCUR compared to the CG (P80 = 0.000, P40 = 0.000, P20 = 0.004) and the SCG (*p* = 0.000). While among the doses of 20, 40, and 80 mg/kg of NCUR compared to FLU, only 20 mg/kg dose showed a significant difference in reducing immobility time (P20 = 0.016).

### 3.4 Effects of NCUR on the OFT

**FIGURE 5:**
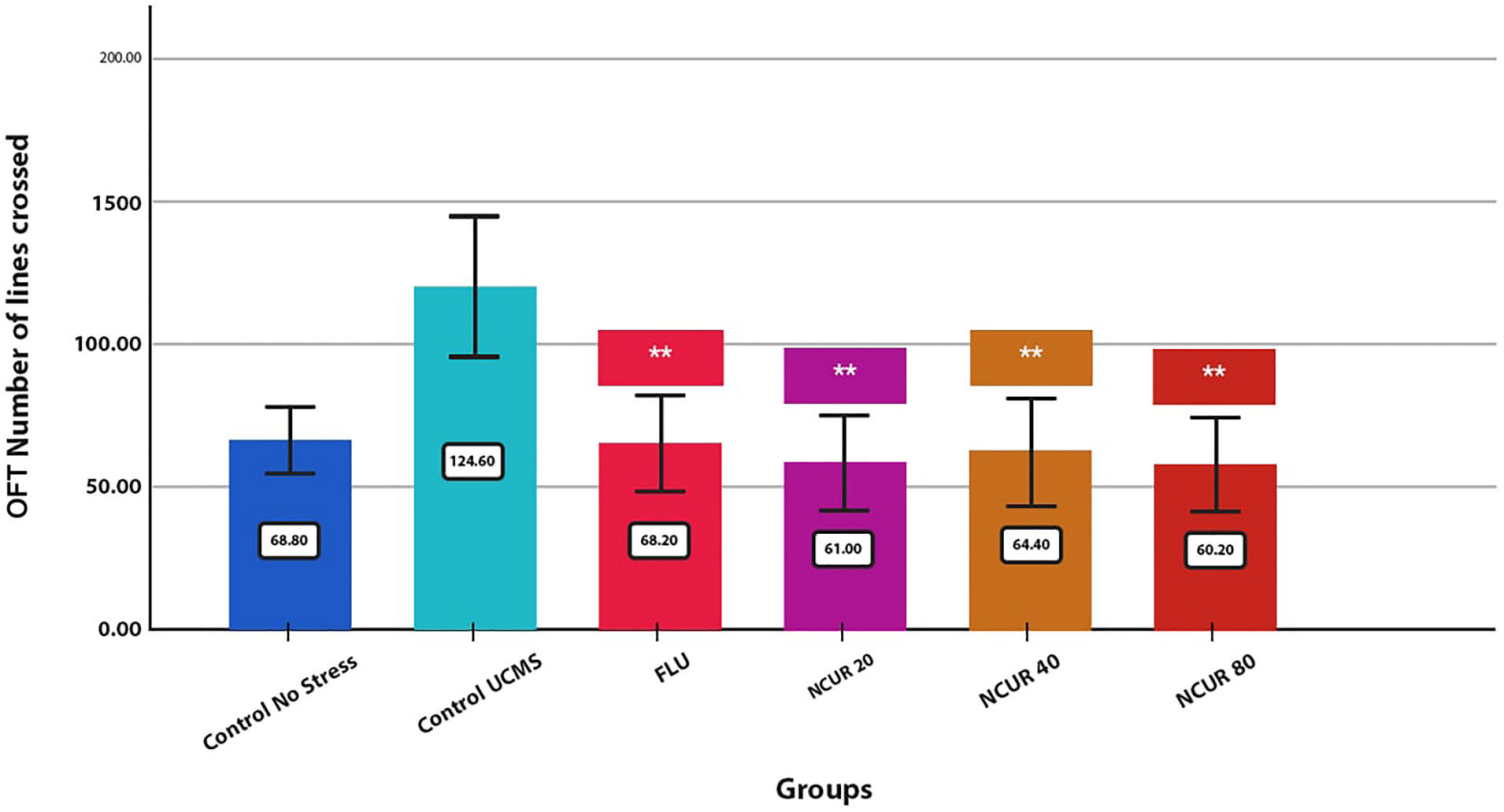
The effect of different doses of NCUR compared to FLU in the OFT. Each group (*N* = 5) was subjected to 26 days of oral gavage. *p* < 0.05 is indicated by #, and *p* < 0.001 is indicated by ## (comparing the CG with other groups). *p* < 0.05 is shown with * and *p* < 0.001 with ** (comparing the SCG with other groups). *p* < 0.05 is shown with $, and *p* < 0.001 is shown with $$ (comparison of FLU with other groups).

The OFT results are demonstrated in Figure 5. Several indicators of depression‐like behavior, including total distance traveled (centimeters) and bouts (count), reflected open‐field behavior in the UCMS experiment. The number of crossed lines by the rats was compared between the groups. There was no significant difference between the three doses of NCUR and the CG. However, there was a significant increase in the level of motor activity of the rats *n* compared to the SCG (*p* = 0.000).

### 3.5 Effects of NCUR on Serum Serotonin Levels

**FIGURE 6:**
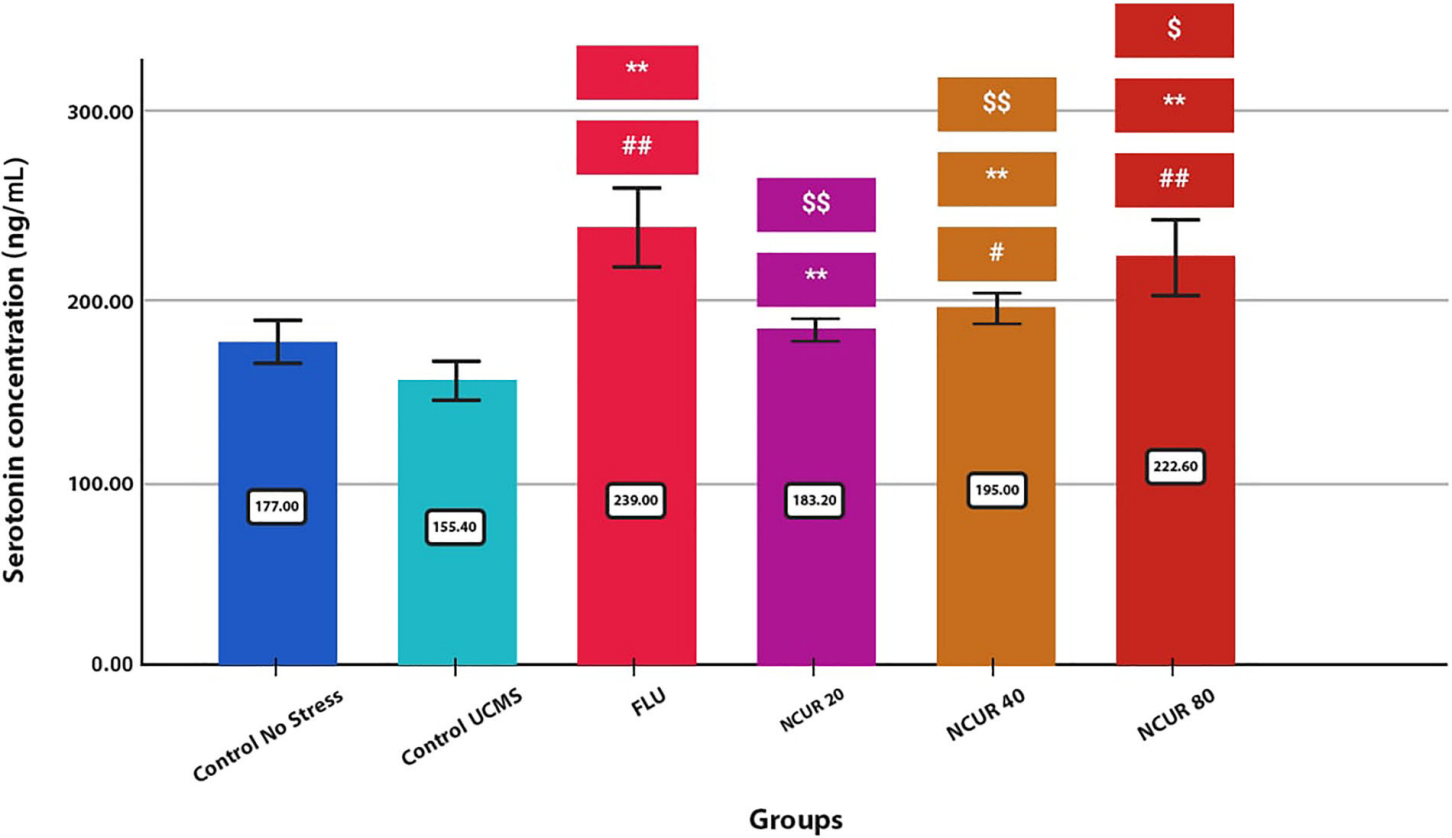
The effect of different doses of NCUR compared to FLU on serum serotonin levels. Each group (*N* = 5) was subjected to 26 days of oral gavage. *p* < 0.05 is indicated by #, and *p* < 0.001 is indicated by ## (Comparing the CG with other groups). *p* < 0.05 is shown with * and *p* < 0.001 with **(Comparing the SCG with other groups). *p* < 0.05 is shown with $ and *p* < 0.001 is shown with $$ (Comparison of FLU with other groups).

All of the doses of NCUR showed a significant difference compared to the CG (P80 = 0.000, P40 = 0.019, P20 = 0.394) and the SCG (P80 = 0.000, P40 = 0.000, P20 = 0.001). Among the doses of NCUR, compared to FLU, all prescribed doses showed a significant increase in serum serotonin levels (P80 = 0.031, P40 = 0.000, P20 = 0.000) (Figure 6).

### 3.6 Effects of NCUR on Serum BDNF Levels

**FIGURE 7:**
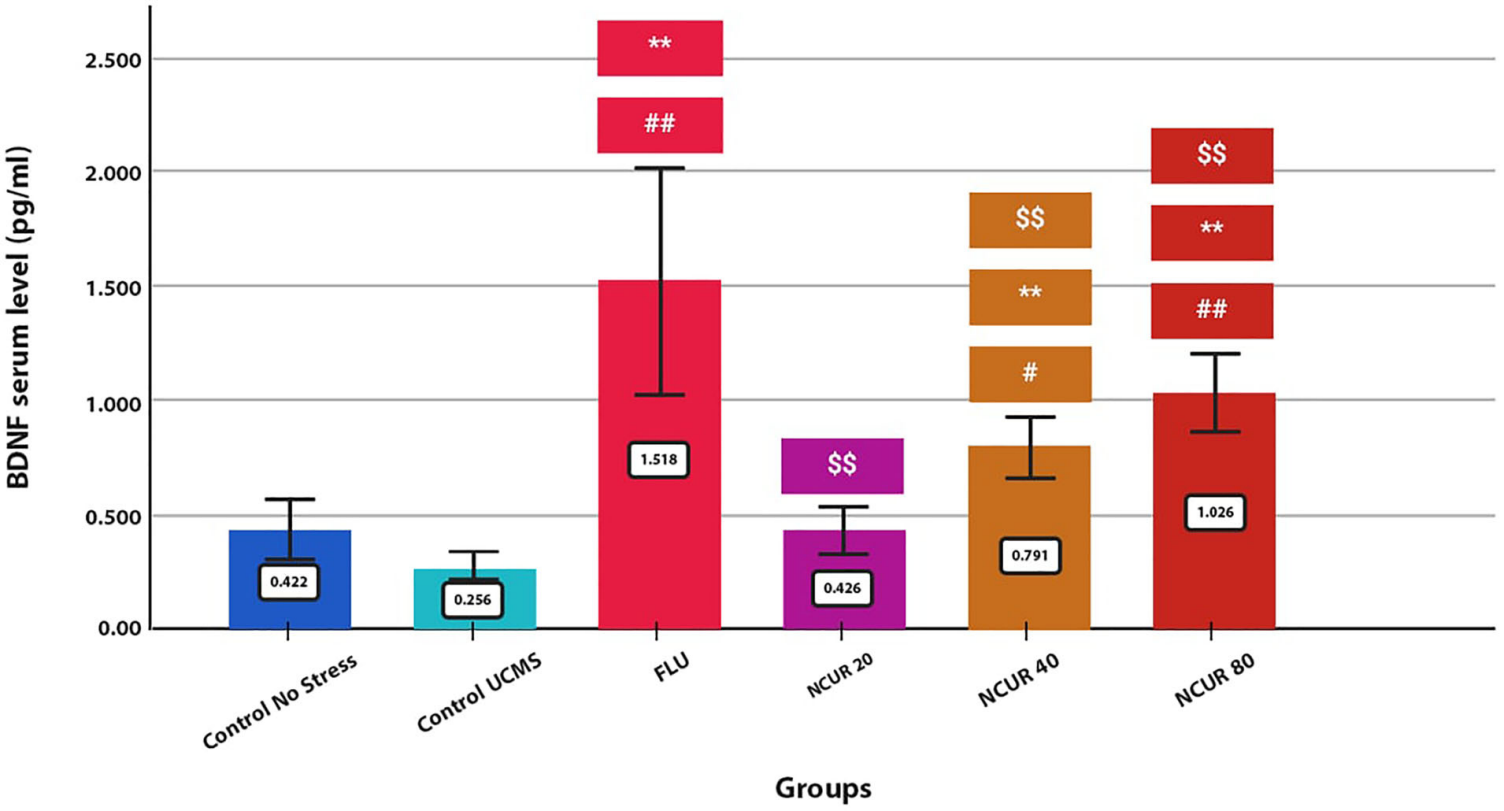
The effect of different doses of NCUR compared to FLU on serum BDNF levels. Each group (*N* = 5) was subjected to 26 days of oral gavage. *p* < 0.05 is indicated by #, and *p* < 0.001 is indicated by ## (comparing the CG with other groups). *p* < 0.05 is shown with * and *p* < 0.001 with **(comparing the SCG with other groups). *p* < 0.05 is shown with $, and *p* < 0.001 is shown with $$ (comparison of FLU with other groups).

The statistical analysis results show a significant difference between the groups regarding BDNF serum levels (Figure 7). All of the doses of NCUR significantly increased the BDNF levels compared to the CG (P80 = 0.000, P40 = 0.005, P20 = 0.974, respectively). However, 40 and 80 mg doses significantly increased the serum level of BDNF compared to the SCG (*p* = 0.000). Also, all the doses demonstrated a significant difference in the increase of serum BDNF compared to FLU (*p* = 0.000).

## 4 Discussion

The most common stress paradigm used to investigate depression is one that exposes subjects to conditions of uncontrollable stress in order to detect antidepressant drug responses. There are several ways to induce depression in rats. These methods include acute (e.g., forced swimming stress, tail suspension stress, and sleep deprivation stress) and chronic (e.g., unexpected chronic stress, restraint for 20–56 days, administration of reserpine, corticosterone, and lipopolysaccharide) exposure to stress (Planchez et al. 2019; Hao et al. 2019). In the current study, the UCMS method was used, which creates the closest model of depression to human depression in rodents (Hao et al. 2019). It was previously stated that chronic unpredictable stress reduced neurogenesis in the brain of rats and resulted in depression‐like symptoms (Mishra et al. 2021), which was also confirmed by the results of our study, which resulted in the development of depression‐like behaviors within 26 days. Also, UCMS has been associated with decreased struggling time in FST (Bogdanova et al. 2013) and decreased exploring activity in OFT (Katz et al. 1981). In this study, exposure to UCMS for 26 days significantly induced anxiety and depressive‐like behaviors, as indicated by the decrease in the sucrose preference index, the increase in immobility in FST and TST, and the increase in the number of bouts in OFT.

Antidepressants have been reported to reverse depression‐like symptoms induced by UCMS (Mutlu et al. 2012). According to previous studies (Lopresti et al. 2014; Sanmukhani et al. 2014; Esmaily et al. 2015; Lopresti and Drummond 2017; Bergman et al. 2013; Yu et al. 2015), curcumin administration in humans exhibits antidepressant‐like effects; however, its low bioavailability keeps it from reaching the therapeutic threshold (Tsai et al. 2011). The current study used NCUR instead of curcumin to investigate its effects on depressive‐like behaviors. The present study demonstrated that the administration of NCUR exhibited antidepressant‐like activities in the UCMS‐induced depression model. Chronic administration of NCUR normalized behavioral changes (SPT, FST, TST, OFT) in rats exposed to stress.

In a study by Wu et al., a similar method was used to investigate the effects of curcumin on depression in 2021 (Wu et al. 2021). The UCMS interval in their study was 6 weeks, whereas the duration of depression‐like behaviors in the current study was shorter (26 days). In this study, we were able to induce depressive‐like behaviors in rats, as compared with their study, and we obtained similar results in our behavioral tests as they had in their study. The results of the present study showed that chronic administration of NCUR had a substantial effect on controlling the behavioral changes that had been caused by UCMS and could reverse the underlying complications.

As the brain's most abundant neurotrophin, BDNF plays a critical role in neural survival as well as synaptic plasticity (Nobis et al. 2020). In previous studies, BDNF was investigated as a potential candidate for causing the antidepressant effects of curcumin (Hurley et al. 2013; Wu et al. 2021). In rats exposed to UCMS, the expression of BDNF was significantly increased following chronic administration of curcumin (Wu et al. 2021). These results correlate with those of this study. The present study provides evidence supporting the link between NCUR's antidepressant‐like properties and levels of BDNF and serotonin in the blood serum. In this study, we observed that the chronic administration of NCUR normalized behavioral changes in rats exposed to UCMS, showing the antidepressant activity of NCUR. Furthermore, we found that by increasing the dosage of NCUR, we could obtain better results on behavioral tests.

Many animal studies suggested that curcumin could reverse depressive‐induced behaviors with many mechanisms such as an increase in BDNF levels in the hippocampus (Hurley et al. 2013), extracellular signal regulated kinase (ERK) phosphorylation and increased BDNF expression in the amygdala (Zhang et al. 2012), activation of the PGC‐1α/FNDC5/BDNF pathway (Wu et al. 2021), preventing the decrease in the expression of synapse‐related proteins such as BDNF, postsynaptic density protein (PSD‐95), and synaptophysin (Zhang et al. 2014), raising the serotonin levels in the brain by upregulating tryptophan hydroxylase‐2 and 5‐hydroxytryptamine1A,2A receptor messenger RNA (mRNA), and downregulating monoamine oxiadase A (MAOA) mRNA in the limbic system and with upregulation of aromatase, BDNF mRNA, and EKR 1 and2 protein in the limbic system, relative to FLUand estradiol (Abd‐Rabo et al. 2019). Our study was in line with these studies, which demonstrated an increase in BDNF and serotonin levels of serum in comparison with FLU.

In the current study, the antidepressant‐like effects of NCUR resulted in a rise in BDNF and serotonin levels in serum, which could serve as a promising strategy for its use as a supplement along with other antidepressants during depression. Curcumin combined with escitalopram or venlafaxine was studied earlier; however, the results were contradictory. Bergman et al. (2013) found that there was no conclusive evidence that there was any benefit in combining curcumin with these drugs. In contrast, Yu et al. (2015) found a synergistic effect of curcumin when combined with escitalopram.

This study has several limitations that should be addressed in future research to deepen the understanding of NCUR's therapeutic potential. First, while this study focused on behavioral responses and serum markers (BDNF and serotonin) to assess NCUR's effects, it did not include neurochemical analyses in specific brain regions, such as the hippocampus and amygdala. Examining these areas could shed light on the neural mechanisms underlying NCUR's effects on depression. Second, cortisol, a primary biomarker of stress and commonly elevated in depression, was not measured in this study. Chronic stress paradigms, like UCMS, often increase plasma cortisol levels, which can reveal valuable insights into physiological stress responses and their modulation by NCUR In addition, cortisol analysis in future studies could, therefore, strengthen the evidence for NCUR's impact on the stress‐depression continuum, enhancing the study's translational relevance. Lastly, although prior research suggests the safety of curcumin and its nanoformulations, this study did not conduct toxicity testing. Incorporating these neurochemicals, biochemical, and safety assessments in future work would enhance the findings of depth and translational relevance, supporting NCUR's potential as a therapeutic supplement.

Future studies should also explore the dose–response relationship of NCUR to determine the optimal therapeutic dosage. Additionally, long‐term studies are necessary to assess the sustainability of NCUR's antidepressant effects and to monitor any potential delayed adverse effects. Investigating the effects of NCUR in combination with standard antidepressant therapies could provide insights into potential synergistic effects, offering a more comprehensive approach to depression treatment.

## 5 Conclusion

In conclusion, our data showed that the administration of NCUR would inhibit UCMS‐induced depression, which would result in an increase in BDNF and serotonin levels. Based on the results obtained, it can be said that increasing the dosage of NCUR would yield better results and it is possible to use it at par with FLU. There is, however, a need for more studies to be conducted in order for NCUR to be used in clinics.

## Author Contributions

All authors contributed equally to the design, execution, analysis, and writing of this study.

## Conflicts of Interest

The authors declare no conflicts of interest.

### Peer Review

The peer review history for this article is available at https://publons.com/publon/10.1002/brb3.70320.

## Data Availability

The data that supports the findings of this study are available upon request from the corresponding author, however are not publicly available as they are currently provided only for the review process.
