# Correction to “Evaluation of Nanocurcumin Effects on Depressive‐Like Behaviors in Rats and Determination of Serum BDNF and Serotonin Levels”

**DOI:** 10.1002/brb3.70523

**Published:** 2025-05-19

J. M. Hadipour, H. Charousaei, and A. Charousaei. “Evaluation of Nanocurcumin Effects on Depressive‐Like Behaviors in Rats and Determination of Serum BDNF and Serotonin Levels,” *Brain and Behavior* 15, no. 2 (2025): e70320.

In the published version of this article, the affiliation of the first author, Mahsa Hadipour Jahromy, was listed incorrectly (It appears that the original listing used the surname “Jahromi” instead of the correct “Jahromy”).

The correct affiliation is:

Herbal Pharmacology Research Center, Faculty of Medicine, Islamic Azad University, Tehran Medical Sciences, Tehran, Iran

We apologize for this error.

